# Remarkably high tensile strength and lattice thermal conductivity in wide band gap oxidized holey graphene C_2_O nanosheet

**DOI:** 10.1186/s11671-024-04046-0

**Published:** 2024-06-11

**Authors:** Fazel Shojaei, Qinghua Zhang, Xiaoying Zhuang, Bohayra Mortazavi

**Affiliations:** 1 Department of Chemistry, Faculty of Nano and Bioscience and Technology, Persian Gulf University, Bushehr, 75169 Iran; 2 Institute of Photonics, Department of Mathematics and Physics, Leibniz Universität Hannover, Welfengarten 1A, 30167 Hannover, Germany; 3 Cluster of Excellence PhoenixD, Leibniz Universität Hannover, Welfengarten 1A, 30167 Hannover, Germany

**Keywords:** Oxidized holey graphene, Semiconductor, Machine learning, Tensile strength, Thermal conductivity

## Abstract

Recently, the synthesis of oxidized holey graphene with the chemical formula C_2_O has been reported (J. Am. Chem. Soc. 2024, 146, 4532). We herein employed a combination of density functional theory (DFT) and machine learning interatomic potential (MLIP) calculations to investigate the electronic, optical, mechanical and thermal properties of the C_2_O monolayer, and compared our findings with those of its C_2_N counterpart. Our analysis shows that while the C_2_N monolayer exhibits delocalized π-conjugation and shows a 2.47 eV direct-gap semiconducting behavior, the C_2_O counterpart exhibits an indirect gap of 3.47 eV. We found that while the C_2_N monolayer exhibits strong absorption in the visible spectrum, the initial absorption peaks in the C_2_O lattice occur at around 5 eV, falling within the UV spectrum. Notably, we found that the C_2_O nanosheet presents significantly higher tensile strength compared to its C_2_N counterpart. MLIP-based calculations show that at room temperature, the C_2_O nanosheet can exhibit remarkably high tensile strength and lattice thermal conductivity of 42 GPa and 129 W/mK, respectively. The combined insights from DFT and MLIP-based results provide a comprehensive understanding of the electronic and optical properties of C_2_O nanosheets, suggesting them as mechanically robust and highly thermally conductive wide bandgap semiconductors.

## Introduction

Nanoporous graphene lattices, in recent years have garnered significant attention in both scientific research and industrial applications due to their appealing physical and chemical properties and versatile capabilities. Graphene [1–3], the two-dimensional (2D) form of sp^2^ carbon atoms, is renowned for its exceptional mechanical strength and flexibility [4], ultrahigh thermal conductivity [5, 6], and intriguing electronic and optical properties [7–10], however is a semimetal and thus lacks a semiconducting electronic nature. This inherent electronic characteristic poses a limitation on the graphene applications, as the presence of an appropriate electronic band gap is crucial for most advanced applications in nanoelectronics, optoelectronics, and catalysis. Introducing ordered porosities into graphene, like making graphene-kirigami [11], -nanomesh [12], antidot [13, 14] or nanoporous graphene [15, 16], have been already employed to develop an appropriate band gap in the electronic structure of graphene [17–19], which have been proven to boost efficiency for diverse chemical processes [20–22] and other advanced technologies, like the DNA sequencing [23–25]. In addition to the semiconducting electronic nature, another key advantage of nanoporous graphene lattices lies in their high surface area to volume ratio, providing abundant active sites for various chemical reactions, adsorption processes, making them as more efficient candidate for applications such as catalysis, gas separation, and energy storage. In 2015, the wet-chemical reaction technique enabled the fabrication of a nanoporous C_2_N lattice with semiconducting electronic properties [26]. Worth noting that because of the existence of the covalent interactions in nanoporous graphene lattices, they are expected to show remarkably high mechanical strength, lower than that of the graphene, but comparable, resulting in remarkable structural stability.

In a latest breakthrough in the synthesis of nanoporous carbon-based nanomembranes, an oxidized holey graphene with a chemical formula of C_2_O, was fabricated through an irreversible nucleophilic aromatic substitution reaction [27]. From a practical standpoint, assessing the various physical properties of the C_2_O nanosheets is crucial to determine their suitability across diverse applications. While the electronic and optical properties of 2D materials are usually key features for their applications, a thorough examination of their thermal and mechanical properties is likewise important. Due to the critical importance of thermal management in nanoelectronics [28–30], semiconducting nanosheets with higher thermal conductivity are particularly desirable. Additionally, mechanical properties are vital as they determine the stability and flexibility of nanomembranes under mechanical loads during operation, directly impacting overall durability of nanodevices. To efficiently explore the aforementioned properties, we employed a synergistic approach, combining first-principles density functional theory (DFT) calculations with classical modeling based on machine learning interatomic potentials (MLIPs). This comprehensive analysis allowed us to evaluate the structural, electronic, optical, mechanical, thermal expansion, and lattice thermal conduction properties of the pristine and suspended C_2_O monolayer.

## Computational methods

The Vienna Ab-initio Simulation Package (VASP) [31, 32] was utilized, employing the generalized gradient approximation (GGA) and PBE exchange correlation functional [32] and DFT-D3 [33] van der Waals (vdW) dispersion for different computations, including the structural optimizations, ab-initio molecular dynamics (AIMD) simulations, analyses of mechanical properties, and electronic structure calculations. As discussed in our previous study [34], the inclusion of DFT-D3 [33] vdW dispersion correction can enhance the accuracy of the modeling results for nanoporous nanosheets. The plane wave cutoff energy was set as 500 eV, while the self-consistency convergence criterion energy was set at 10^–4^ and 10^–6^ eV, for geometry optimizations and analysis of electro-optical properties, respectively. In order to achieve geometry optimization and stress-free structures, adjustments were made to atomic positions and lattice sizes by using the conjugate gradient algorithm until the Hellman–Feynman forces on each atom reduced to below 0.002 eV/Å on each atom. The nanosheets were positioned in the XY-plane and in order to remove interactions between neighboring cells in the Z direction, around 15 Å vacuum space was introduced. The deformation potential theory was utilized to estimate the carrier mobilities limited by acoustic phonon scattering in C_2_N and C_2_O monolayers. The carrier mobilities were calculated using the formula: $\frac{{{\text{e}}\hbar^{3} C_{2D} }}{{{\text{KT}}\left( {m_{e}^{*} } \right)^{2} \left( {E_{i} } \right)^{2} }}$, where, K is the Boltzmann constant, ℏ represents the reduced Planck’s constant, $C_{2D}$ and $m_{e}^{*}$ stand for the elastic modulus and the effective mass of the carrier along the transport direction, respectively. $E_{i}$ signifies the deformation potential constant of the carrier due to the acoustic phonons for the i-th edge band along the transport direction ($E_{i}$ = $\frac{{\Delta E_{i} }}{{\Delta l/l_{0} }}$). Here, $\Delta E_{i}$ represents the change in the absolute energy position of *i*th edge band, $l_{0}$ is the lattice constant along the transport direction, and $\Delta l$ is the deformation of $l_{0}$. The light absorption properties were analyzed by calculating their frequency-dependent dielectric matrix, by neglecting the local field effects, on the basis of the random phase approximation, using the same methodology as our previous works [34, 35].

A moment tensor potential (MTP) [36] was fitted to investigate the structural, dynamical, thermal and mechanical properties of the C_2_O monolayer, using the MLIP package [37]. The training dataset was prepared by the AIMD calculations over the stress-free and stretched unitcells under varying temperatures, using the same methodology of our recent works [38, 39], adopting a time step of 1 fs, NVT ensemble, DFT-D3 correction and 2 × 2 × 1 K-point grid. The complete AIMD datasets with 3200 configurations was equally subsampled to 640, and an MTP with a cutoff distance of 3.5 Å was trained using the two-step passive training approach [40].The phonon dispersion relation was obtained by utilizing the refined MTP, employing 3 × 3 × 1 supercells and applying the small displacement technique from the PHONOPY package [41], as detailed in our previous study [42]. VESTA [43] and OVITO [44] free packages were employed to illustrate the atomic structures.We utilized the LAMMPS package [45] to examine thermal and mechanical properties based on the trained MTP, with a time step of 0.5 fs. We assumed a fixed thickness of 3.5 Å for the C_2_N and C_2_O monolayers, according to the experimental report [27]. Non-equilibrium molecular dynamics (NEMD) simulations on the basis of the trained MTP were carried out to evaluate the length-dependent lattice thermal conductivity of the C_2_O monolayer, using the same approach as that discussed in our previous studies [46–48].

## Results and discussion

**Fig. 1:**
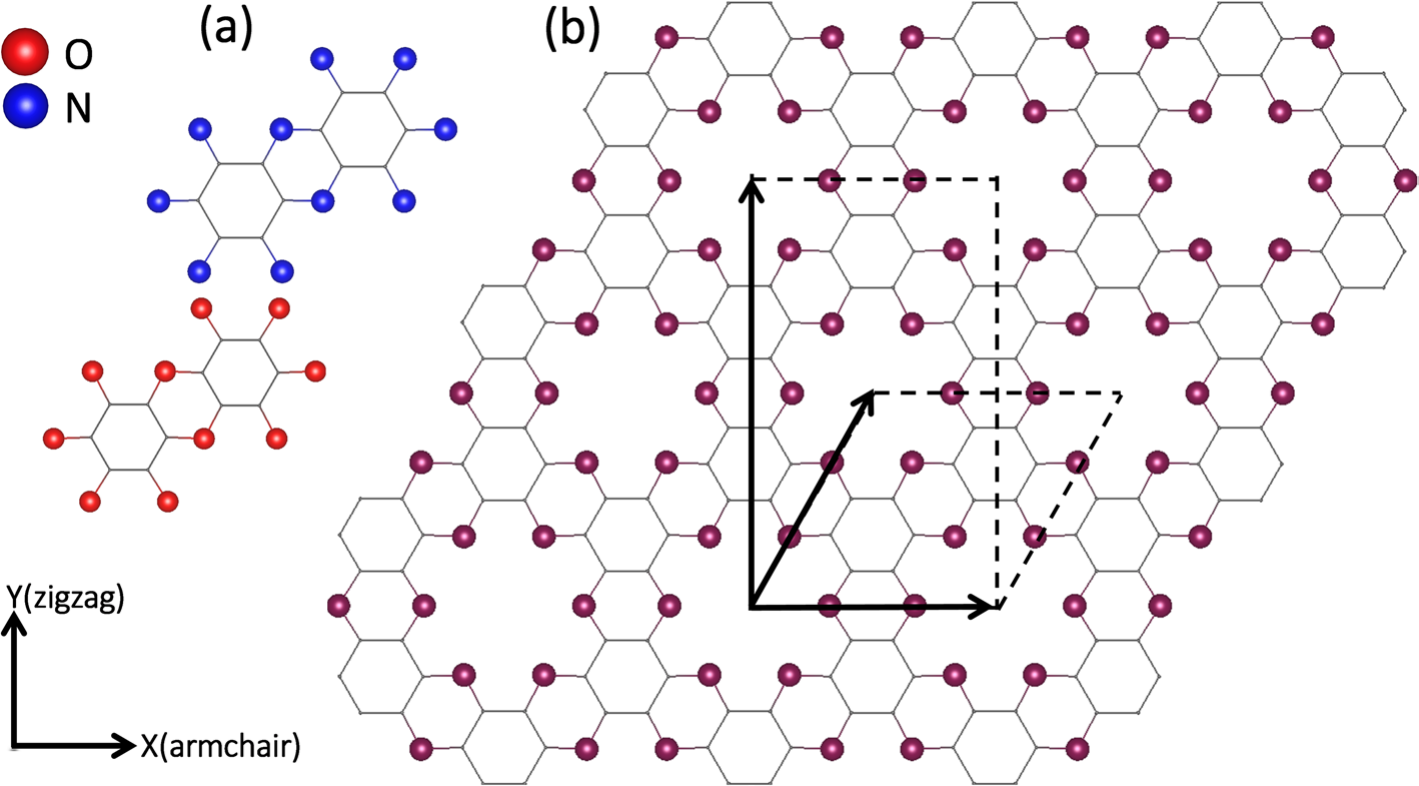
(**a**) Constructing molecules and (**b**) top view of crystal structures of C_2_O and C_2_N monolayers. Both the hexagonal primitive cell and the smallest rectangular cell of each one are also shown. The most stable structures appear completely planar. The thin gray lines in this figure represent carbonaceous backbone of the C_2_X monolayers

Figure 1 illustrates the top view of the crystal structure of the extensively studied C_2_N monolayer. The C_2_N monolayer features a topologically flat structure characterized by a hexagonal primitive cell and the symmetry group P6/MMM (No. 191). Despite having different conjugation characteristics, the C_2_O monolayer shares a similar crystal structure with the C_2_N counterpart. The two monolayers can be envisioned as benzene rings covalently bonded together within the same plane, facilitated by doubly coordinated nitrogen or oxygen atoms, resulting in the formation of flat structures. The resulting networks are porous, and in the case of C_2_O, the pores resemble the structure of graphene embedded 18-Crown-6 ether [49]. In order to investigate the comparative synthesis favorability of the C_2_O and C_2_N monolayers, we calculated their polymerization energy. According to an experimental report [27], the C_2_O lattice was simply synthesized by the self-condensation reaction of trichlorophloroglucinol: 2C_6_O_3_Cl_3_H_3_ → 6C_2_O + 6HCl. While there are several synthesis routes for the synthesis of C_2_N [50, 51], we adopted the condensation reaction between hexaaminobenzene and hexaketocyclohexane, resulting in: C_6_N_6_H_12_ + C_6_O_6_ → 6C_2_N + 6H_2_O. To calculate the polymerization energies of the C_2_N and C_2_O monolayers, we employed the following formulas:

For the C_2_N monolayer:

1 $${\text{E}}_{{\text{p}}} \left( {{\text{C}}_{{2}} {\text{N}}} \right) = {6} \times {\text{E}}_{{{\text{tot}}}} \left( {{\text{C}}_{{2}} {\text{N}}} \right) + {6} \times {\text{E}}_{{{\text{tot}}}} \left( {{\text{H}}_{{2}} {\text{O}}} \right){-}{\text{E}}_{{{\text{tot}}}} \left( {{\text{C}}_{{6}} {\text{O}}_{{6}} } \right){-}{\text{E}}_{{{\text{tot}}}} \left( {{\text{C}}_{{6}} {\text{N}}_{{6}} {\text{H}}_{{{12}}} } \right)$$

For the C_2_O monolayer:

2 $${\text{E}}_{{\text{p}}} \left( {{\text{C}}_{{2}} {\text{O}}} \right) = {6} \times {\text{E}}_{{{\text{tot}}}} \left( {{\text{C}}_{{2}} {\text{O}}} \right) + {6} \times {\text{E}}_{{{\text{tot}}}} \left( {{\text{HCl}}} \right){-}{2} \times {\text{E}}_{{{\text{tot}}}} \left( {{\text{C}}_{{6}} {\text{O}}_{{3}} {\text{Cl}}_{{3}} {\text{H}}_{{3}} } \right)$$

In these equations, E_tot_(C_2_N) and E_tot_(C_2_O) represent the total electronic energies of a formula unit of the C_2_N and C_2_O monolayers, respectively. All other terms correspond to the total energies of their isolated precursor molecules. The process for the C_2_N nanosheet formation is found to be 4.01 eV exothermic, whereas that for the C_2_O counterpart is by 1.85 eV endothermic, indicating that the C_2_N monolayer formation is thermodynamically more favorable. Shifting our focus to the structural properties of the two monolayers, the optimized lattice constants for the C_2_O and C_2_N monolayers are 8.24 and 8.33 Å, respectively. The calculated lattice constant for the C_2_N is in good agreement with previous theoretical reports (8.325 Å [52] and 8.33 Å [53]), and that of the C_2_O monolayer is also very close to the experimentally determined value of 8.25 Å [27]. Examination of bond lengths in both C_2_O and C_2_N monolayers reveal that despite of the slightly longer lattice constant in the C_2_N lattice, C–C bond lengths in C_2_N (1.43 and 1.47 Å) are slightly more elongated than their counterparts in the C_2_O lattice (1.39 and 1.40 Å). This preliminary observation suggests that the C_2_O sheet may exhibit higher mechanical strength compared to the C_2_N lattice. Moreover, the C–C bond lengths in C_2_O (1.39 and 1.40 Å) are almost identical to those found in isolated benzene molecules (1.39 Å [54]). Notably, the C-O bonds in C_2_O are single bonds with a bond length of 1.38 Å, while the C–N bonds in C_2_N are partial double bonds with a bond length of 1.33 Å. These observations in bond lengths in C_2_O and C_2_N monolayers can be explained by considering their different π-conjugation characteristics. In the C_2_N structure, each nitrogen atom exhibits sp^2^ hybridization. This configuration allows one electron to contribute to the π-system of the adjacent ring, while the nitrogen's lone pair remains in the plane of the sheet, localized within the pore. In the C_2_O nanosheet, however, oxygen atoms and their lone pairs adopt a tetrahedral configuration due to sp^3^ hybridization. This difference in hybridization between nitrogen and oxygen atoms in the respective monolayers results in distinct electronic properties. The C_2_N monolayer features delocalized π-conjugation, arising from a network of fused aromatic rings. In contrast, the C_2_O counterpart contains oxygen-linked rings that interrupt the aromaticity between benzene units, thus confining π-conjugation to the individual benzene rings. The difference in π-conjugation characteristics between the two systems is expected to yield distinct physico-chemical and electronic properties in each. The difference in π-conjugation characteristics between the two systems is expected to yield distinct physico-chemical and electronic properties in each.

**Fig. 2:**
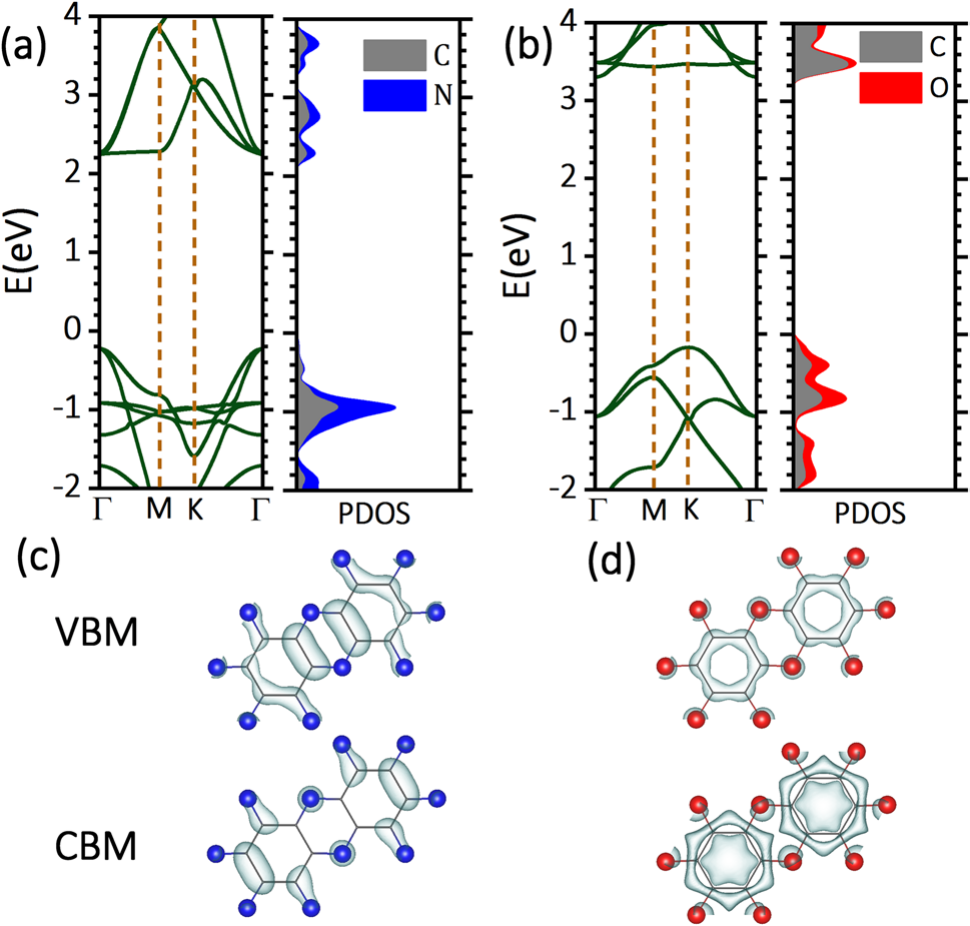
HSE06 band structure alongside partial and projected density of states (PDOS) (**a**, **b**), as well as the charge density distribution of the valance band maximum (VBM) and conduction band minimum (CBM) (**c**, **d**) of each of the C_2_N to C_2_O monolayers. Fermi level is set to 0 eV

Replacing all nitrogen atoms in C_2_N monolayer with oxygen atoms, introduces six additional electrons into each primitive cell, while also disrupting the π-conjugation. These dual alterations are anticipated to induce significant changes in the electronic structure from C_2_N to C_2_O. To investigate the electronic structures of C_2_O and C_2_N monolayers, we conducted band structure calculations using both PBE and HSE06 functionals. In Fig. 2, we present the HSE06 band structure alongside partial and projected density of states (PDOS), as well as the charge density distribution of the valance band maximum (VBM) and conduction band minimum (CBM) of each of C_2_N to C_2_O nanosheets. Based on the data depicted in Fig. 2, single layer C_2_N emerges as a direct gap semiconductor, exhibiting an HSE06 (PBE) band gap of 2.47 (1.67) eV at the Γ point. This band gap value agrees with previous reports (2.46 and 2.47 eV) [55–57]. The corresponding band gap in the C_2_O monolayer increases to 3.47 (2.23) eV, with the VBM shifting to the K point, resulting in an indirect band gap. The increased band gap in C_2_O, compared to C_2_N, is likely attributed to the disruption of delocalized π-conjugation upon substituting nitrogen atoms with oxygen atoms. Although there are differences in their band structures, both C_2_N and C_2_O monolayers exhibit dispersed valence and conduction bands, suggesting potentially high carrier mobilities for both systems. To gain further insights into the electronic structure of C_2_N and C_2_O, we conducted a detailed analysis of their PDOS and charge density distribution of their VBMs and CBMs. Based on the data presented in Fig. 2c, both VBM and CBM of C_2_N exhibit delocalized π-characteristics. In the case of C_2_O, as shown in Fig. 2d the VBM also demonstrates delocalized π-characteristics, albeit it can be conceptualized as an antibonding interaction between the lowest energy π-molecular orbital of a benzene ring and the purely p_z_-orbitals of the surrounding oxygen atoms, as per the crystal orbital bond index (COBI) analysis conducted using LOBSTER [58]. The occupation of such antibonding states leads to the disruption of π-conjugation within the C_2_O layer by breaking down the π bond between carbon and oxygen atoms. This form of orbital hybridization aids in avoiding anti-aromaticity, and consequently, instability in the oxygen-containing rings between benzene units. Further confirmation is provided by observing that the hydrogenation of pore nitrogen atoms in the C_2_N sheet results in nearly identical dispersion patterns for the three top valance bands as in the C_2_O counterpart. It is noteworthy that hydrogenated C_2_N is isoelectronic with C_2_O. In fact, both materials avoid the anti-aromaticity of the linker ring at the expense of sacrificing delocalized π-conjugation. The CBM, however, represents an in-plane σ state, indicative of an anti-bonding interaction between the benzene ring and its surrounding oxygen. The distinct nature of band edge states in C_2_N and C_2_O monolayers may lead to varying electronic behaviors in response to in-plane strains.

Carrier mobility is a fundamental parameter in semiconductor physics, defining the speed at which charge carriers move under an electric field. This parameter directly impacts the performance of electronic devices based on 2D materials, from transistors to optoelectronic devices, making it essential for advancing nanoelectronics and next-generation technologies. Mobility is profoundly constrained by scattering phenomena within materials, with lattice vibrations constituting a primary source. These scattering events, arising from lattice vibrations, govern intrinsic mobility and establish the upper limit for defect-free 2D materials. We utilized the deformation potential theory (DPT) method to assess the intrinsic charge carrier mobility within the C_2_N and C_2_O monolayers. It is worth noting that a prior study found that unlike many other 2D materials, the electron mobility in the C_2_N monolayer at room temperature is primarily influenced by optical phonons rather than acoustic phonons [58]. However, the DPT theory we apply in our work to examine carrier mobilities accounts only for scattering from longitudinal acoustic (LA) phonon modes. Despite this limitation, we believe that a comparative analysis of acoustic (LA) phonon-limited mobility in the two systems of the C_2_N and C_2_O nanosheets remains valuable. For computational ease, we adopted the smallest rectangular cell for each monolayer to compute the electron and hole mobilities along in-plane directions (X(armchair) and Y(zigzag) directions). The resulting carrier mobilities and associated quantities using the rectangular cells for the C_2_N and C_2_O monolayers are summarized in Table 1. Analysis of the data in Table 1 reveals that the C_2_N monolayer displays a maximum electron mobility of 47,640 cm^2^  V^−1^ s^−1^, significantly surpassing its maximum hole mobility of 1844 cm^2^ V^−1^ s^−1^. Our calculated electron mobilities are consistent with those previously reported for the acoustic phonon-limited electron mobilities of the C_2_N monolayer (greater than 10^4^ cm^2^ V^−1^ s^−1^) [58]. It is evident that replacing nitrogen atoms with oxygen atoms markedly suppresses the electron mobility to 684 cm^2^ V^−1^ s^−1^. However, despite significant alterations in effective masses and deformation potentials, the hole mobility remains almost intact.

**Table 1:** Elastic modulus (C_2D_), effective mass of electrons and holes ($m_{e}^{*}$, $m_{h}^{*}$) with respect to the rest mass of an electron (m_0_), deformation energy of the CBM and VBM $\left( {\left| {E_{CBM} } \right|,\;\left| {E_{VBM} } \right|} \right)$, and estimated electron and hole mobilties ($\mu_{e}$, $\mu_{h}$) for C_2_N and C_2_O monolayers, calculated using HSE06 method. Electron and hole mobilities are calculated at 300 K

		$C_{2D}$ (N/m)	$m_{e}^{*}$ ($m_{0}$)	$\left| {E_{CBM} } \right|$ (eV)	$\mu_{e}$ (cm^2^ V^−1^s^−1^)	$m_{h}^{*}$ ($m_{0}$)	$\left| {E_{VBM} } \right|$ (eV)	$\mu_{h}$ (cm^2^ V^−1^ s^−1^)
C_2_N	Armchair	157	0.39	0.98	29,551	0.32	7.39	874
	Zigzag	157	0.24	0.98	47,640	0.15	7.43	1844
C_2_O	Armchair	150	0.61	3.82	620	0.97	1.86	1147
	Zigzag	150	0.55	3.83	684	0.71	1.87	1551

**Fig. 3:**
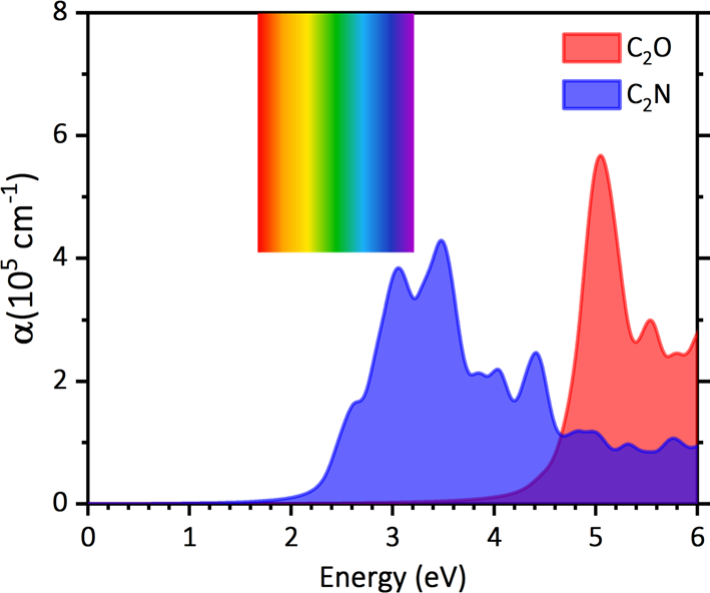
The absorption coefficients of the C_2_N and C_2_O monolayers calculated on the basis of HSE06 functional. The energy range of the visible region of light spectrum is also shown

We next investigate the optical properties of the C_2_N and C_2_O monolayers by calculating their absorption coefficients (α(ω)) using the HSE06-based method. Figure 3 illustrates the absorption coefficients of the C_2_N and C_2_O monolayers under light incidence along the Z-direction and polarization along the zigzag and armchair directions. Since the materials feature isotropic hexagonal lattices, the calculated absorption coefficients remain the same along zigzag and armchair directions. A quick examination of the figure reveals that C_2_N displays notably high absorption coefficients (10^5^ cm^−1^) within the visible region of the light spectrum, in which the first absorption peak appears at 2.62 eV, slightly exceeding its calculated band gap value. In contrast, the first absorption peak of C_2_O occurs at a higher energy of 5.07 eV, significantly surpassing its band gap. Our thorough analysis suggests that the shift of the first absorption peak compared to the band gap, can be attributed to the indirectness of the band gap. In this study, due to the large unit cell of the C_2_O monolayer, excitonic effects have not been incorporated into the analysis of electronic and optical properties, potentially impacting the accuracy of predictions [59–61]. This aspect can be an important topic for oncoming studies.

**Fig. 4:**
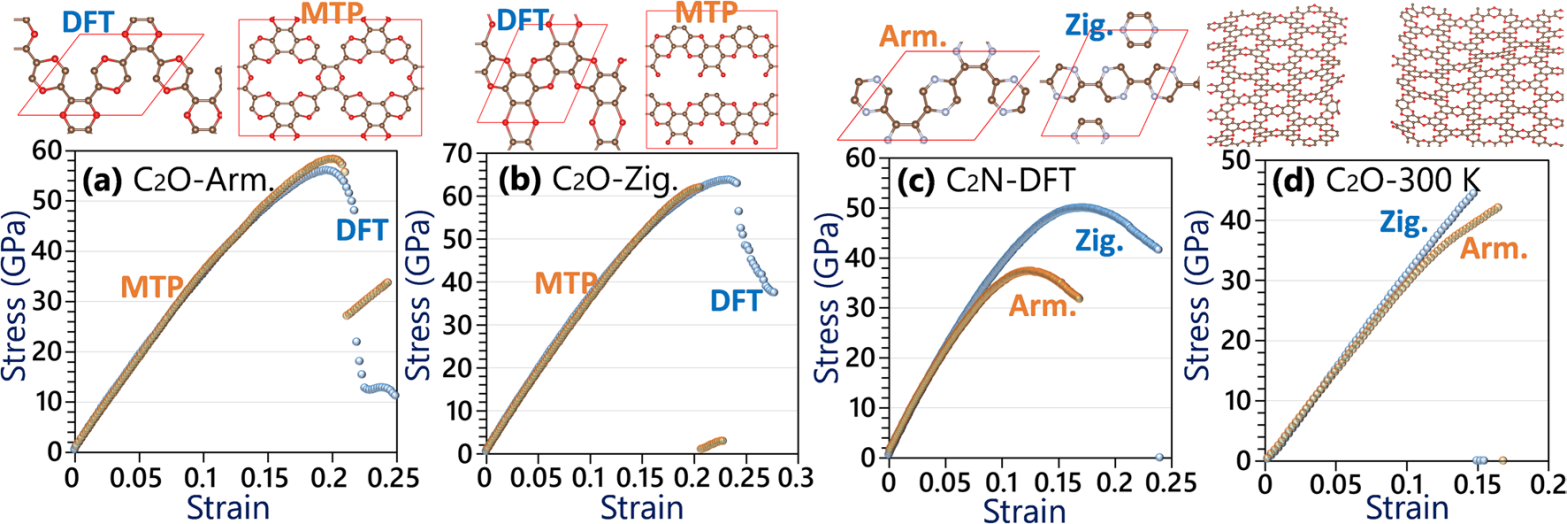
**a**, **b** Predicted uniaxial stress–strain responses of the C_2_O monolayer by the MTP and DFT loaded along the armchair and zigzag directions, respectively. **c** Uniaxial stress–strain response of the C_2_N monolayer by the DFT method. **d** Predicted uniaxial stress–strain curves of the C_2_O monolayer at 300 K. Atomic configurations over every panel illustrate the failure mechanism

We next explore the mechanical/failure responses of the C_2_O monolayers with the DFT and MTP-based model, and compare the obtained results with those of the C_2_N counterpart. The mechanical responses were examined using the uniaxial tensile loading. In this regard within the DFT and MTP-based methods, at the ground state without the temperature effect, the conjugate gradient algorithm coupled with the box-relaxation in the perpendicular direction of loading was employed, to ensure the accurate satisfaction of the uniaxial stress condition. From a modelling point of view, it is crucial that the trained MLIP model accurately reproduces the stress–strain curve up to the ultimate tensile strength point and the failure mechanism as well, which are critical information for the design of nanodevices. As depicted in Fig. 4, the uniaxial stress–strain curves of the C_2_O monolayer predicted by the DFT and the MTP-based model are compared, confirming the remarkable precision of the developed MLIP in scrutinizing the direction-dependent mechanical response of the C_2_O nanosheet. It is useful to highlight that since geometry optimizations were carried out rather than lengthy molecular dynamics calculations, the conducted MTP calculations for a rectangular 2 × 2 × 1 supercell was accomplished with a negligible computational cost, earlier than the completion of the first self-consistent loop of the DFT calculations, using the same computational resource. The tensile strengths of the C_2_O monolayer along the armchair(zigzag) directions are predicted to be 55.8(63.8) GPa, which are distinctly higher than the corresponding values of the 37.4(50.0) GPa predicted for the C_2_N counterpart. As discussed earlier, this is consistent with observed shorter C–C bond lengths in the C_2_O lattice. Failure mechanism analysis shown in Fig. 4 insets, reveals that for the uniaxial loading along the armchair, the failure first happen by homonuclear C–C bond breakages in the central hexagonal rings for both of the C_2_N and C_2_O monolayers. Along the zigzag direction, the rupture however initiates by the breakages of the C-O bonds in the C_2_O monolayers, whereas for the C_2_N counterpart, it happens along the C–C bonds. From the presented results shown in Fig. 4, it can be also concluded that the developed classical model could also precisely reproduce the direction dependent failure mechanism in the C_2_O monolayer. After ensuring the accuracy of the trained MTP, we then evaluate the mechanical properties of the C_2_O nanosheets at 300 K by considering over 1000 atoms. In this case, the quasi-static uniaxial tensile loading was employed, utilizing the Nosé–Hoover barostat and thermostat method (NPT) to satisfy the uniaxial stress conditions, as detailed in our previous studies [38, 39]. The tensile strengths of the C_2_O monolayer at room temperature along the armchair(zigzag) directions are predicted to be 44.4(42.0) GPa, which are close to the value of 42 GPa, reported with MTP for the C_2_N lattice at room temperature [62]. It was nonetheless observed that the failure mechanism stays consistent with that observed at the ground state. The MLIP-based molecular dynamics calculations confirm remarkably high tensile strength of the nanoporous C_2_O monolayer, which is almost half of the that predicted for the pristine graphene [38].

**Fig. 5:**
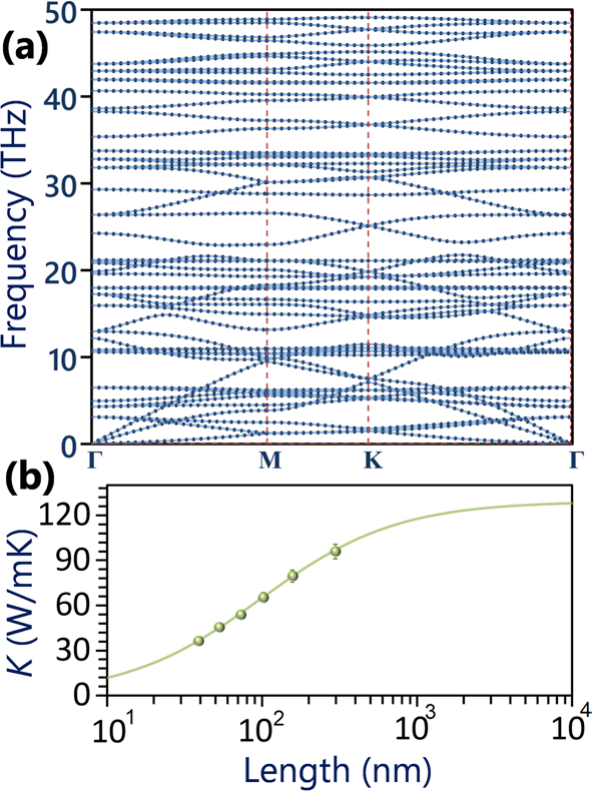
**a** Phonon dispersion relation and **b** length dependent room temperature thermal conductivity of the C_2_O monolayer

We now turn our attention to investigate the lattice thermal conductivity and dynamical stability of the C_2_O nanosheet. The phonon dispersion curve of the C_2_O monolayer predicted using the fitted MTP is shown in Fig. 5a, demonstrating the absence of imaginary frequencies across all three acoustic phonon and optical branches. This observation indicates the desired structural stability of the fully planar configuration of the C_2_O lattice. Originating from the Γ point three acoustic phonon modes initiate, with the out-of-plane mode exhibiting quadratic relationships, while the two in-plane counterparts display linear dispersions, reminiscent of those observed in single-layer graphene and many other 2D materials [42]. In Fig. 5b the MTP-based NEMD predictions for the length effects on the room temperature phononic thermal conductivity of the C_2_O nanosheet is plotted, assuming a thickness of 3.5 Å according to the experimental report [27]. In the NEMD simulations, after the initial equilibration with the NPT method, a few rows of atoms at both ends were fixed and then the remaining of the system was then divided into 22 sections along the heat transport direction, with a 20 K temperature difference applied between the two ends using the NVT method. The remaining part of the system was simultaneously simulated using the constant energy ensemble (NVE) method. Lattice thermal conductivity was determined for each system using 1D Fourier's law, considering the imposed heat flux under NVT conditions and the resulting averaged temperature gradient along the sample's length, as mentioned in our previous studies [46–48]. In consistency with NEMD results for the C_2_N monolayer [57, 63], an increasing trend in the predicted lattice thermal conductivity appears by increasing the nanosheet length up to 300 nm. As a firmly established method, utilizing NEMD predictions for samples of finite lengths $L$ and $\kappa_{L}$, one can estimate the diffusive phononic thermal conductivity *κ*_*∞*_, as a function of the phonons’ mean free path (*Λ*) via [64]:

3 $$\frac{1}{{\kappa_{L} }} = \frac{1}{{\kappa_{\infty } }} \left( {1 + \frac{{\Lambda }}{L}} \right)$$

As depicted in Fig. 5b, based on the fitted line to the NEMD data points using the aforementioned relationship, the room temperature diffusive lattice thermal conductivity of the C_2_O monolayer is estimated to be 129 ± 8 W/mK. This value is noticeably higher than the previous theoretical predictions of the 85 and 82 W/mK for the C_2_N counterpart, respectively, using the NEMD with MTP [62] and Boltzmann transport equation coupled with DFT [65] calculations. The high lattice thermal conductivity and wide band gap of the C_2_O nanosheet, suggest them as promising candidates for the enhancement of the polymeric materials thermal conductivity, particularly appealing for the thermal managements in nanoelectronics.

**Fig. 6:**
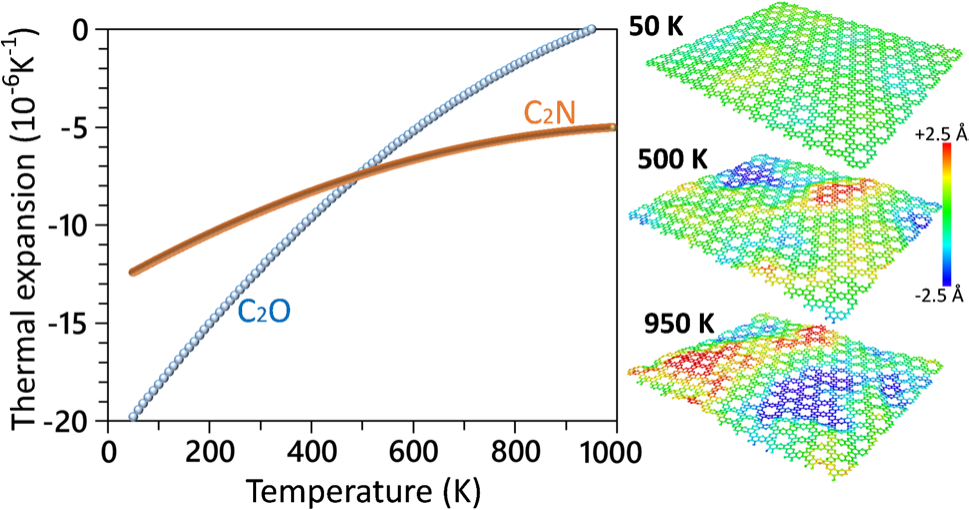
Thermal expansion coefficients of the suspended C_2_O and C_2_N (data taken from Ref. [66]) monolayers as a function of temperature. The side views of the equilibrated C_2_O monolayers at different temperatures are also illustrated, with color coding representing the out-of-plane displacements of atoms with respect to the center of atomic mass

We finally examine the thermal expansion behavior of the C_2_O monolayer using the MTP-based model, using the same methodology as that validated in our previous study [63]. Thermal expansion coefficients were evaluated based on the temperature dependency of the projected area (A) using the $\frac{1}{A}\frac{dA}{{dT}}$ relation [63, 64]. To establish this relationship, we conducted seven independent calculations with uncorrelated initial velocities using the large system with 3528 atoms. The projected areas at various temperatures were averaged, and a polynomial curve was fitted to determine the thermal expansion coefficients [63, 64]. In Fig. 6, the thermal expansion coefficients of the single-layer C_2_O and C_2_N (data taken from Ref. [66]) are compared. At room temperature, the thermal expansion coefficients for the suspended C_2_O and C_2_N monolayers are predicted to be relatively close, − 12.2 and − 9.4 × 10^−6^ K^−1^ [66], respectively. When analyzing the predicted thermal expansion coefficients, it becomes evident that at low temperatures, the thermal expansion coefficients of the C_2_O monolayer are significantly more negative than its C_2_N counterpart. However, as the temperature increases, the negative thermal expansion coefficient of the C_2_O nanosheets decreases more conspicuously than that of the C_2_N, and approaches to the zero value at around 900 K. This observation suggests that at temperatures below 400 K, the contractions along in-plane directions because of the out-of-plane wrinkles formations, are more considerable in the C_2_O monolayer than the C_2_N counterpart. However, as temperature increases, the out-of-plane deflections of the C_2_O nanosheet becomes more limited. The presented results indicate high flexibility of the C_2_O nanomembranes, particularly at low temperatures.

## Concluding remarks

Inspired by the latest synthesis of the oxidized holey graphene with a chemical formula of the C_2_O, through irreversible nucleophilic aromatic substitution reaction [27], herein comprehensive first-principles calculations were carried out to explore the electronic, optical, mechanical, and thermal properties of the single-layer and free-standing C_2_O, and compared the acquired findings with those of the C_2_N monolayer. The comparison of C_2_N and C_2_O nanosheets underscores their unique electronic and optical properties. Delocalized π-conjugation in the C_2_N contributes to its direct band gap and superior electron mobilities. Switching from N to O in the C_2_O monolayer, considerably suppresses the electron mobility but maintains hole mobility. While the C_2_N monolayer exhibits a strong light absorption in the visible spectrum, the initial absorption peaks in the C_2_O lattice occur at around 5 eV, falling within the UV spectrum. Without taking the temperature effects into account, the tensile strengths of the C_2_O monolayer along the armchair(zigzag) directions are predicted to be 55.8(63.8) GPa, which are distinctly higher than the predicted values of 37.4(50.0) GPa for the C_2_N counterpart. It is predicted that the C_2_O nanosheet can exhibit remarkably high tensile strengths over 42 GPa at 300 K. Furthermore, the room temperature lattice thermal conductivities of the C_2_O monolayer is estimated to be 129 ± 8 W/mK, which is also noticeably higher than previous theoretical reports [62, 65] of around 85 W/mK for the C_2_N nanosheet. Analysis of the thermal expansion behavior of the C_2_O monolayer confirms its remarkable thermal flexibility. Presented results, for the first time, highlight the remarkably high tensile strength and thermal conductivity, decent thermal flexibility, strong absorption in UV region of light and wide band gap semiconducting nature of the C_2_O nanosheets, highly promising for applications in nanoelectronics, nanophotonics, polymer nanocomposites and thermal management systems.

## Data Availability

Data that support the findings of this study are available from the corresponding authors upon reasonable request.
